# Analysis of clinical anatomical correlates of motor deficits in stroke by multivariate lesion inference based on game theory

**DOI:** 10.3389/fnins.2025.1409107

**Published:** 2025-04-17

**Authors:** Monica N. Toba, Caroline Malherbe, Melissa Zavaglia, Audrey Arnoux, Mélanie Barbay, Claus C. Hilgetag, Olivier Godefroy

**Affiliations:** ^1^Laboratory of Functional Neurosciences (EA 4559), University Hospital of Amiens, University of Picardie Jules Verne, Amiens, France; ^2^Department of Computational Neuroscience, Hamburg Center of Neuroscience, University Medical Center Hamburg-Eppendorf, Hamburg, Germany; ^3^Department of Neurology, Head and Neuro Center, University Medical Center Hamburg-Eppendorf, Hamburg, Germany; ^4^MIRMI - Munich Institute of Robotics and Machine Intelligence, Technische Universität München, Munich, Germany; ^5^Department of Health Sciences, Boston University, Boston, MA, United States

**Keywords:** game theory, Multi-perturbation Shapley value Analysis (MSA), stroke, lesion inference, motor function, NIHSS

## Abstract

**Introduction:**

The exploration of causal functional inferences on the basis of deficits observed after neurological impairments is often based on the separate study of gray matter regions or white matter tracts. Here, we aimed at jointly analysing contributions of gray matter and white matter by using the domain of motor function and the approach of iterative estimated Multi-perturbation Shapley Analysis (MSA), a multivariate game-theoretical lesion inference method.

**Methods:**

We analyzed motor scores assessed by the National Institute of Health Stroke Scale (NIHSS) together with corresponding lesion patterns of 272 stroke patients using a finely parcellated map of 150 gray matter regions and white matter tracts of the brain.

**Results:**

MSA revealed a small set of essential causal contributions to motor function from the internal capsule, the cortico-spinal tract, and the cortico-ponto-cerebellum tract.

**Discussion:**

These findings emphasize the connectional anatomy of motor function and, on the methodological side, confirm that the advanced multivariate method of iterative estimated MSA provides a practical strategy for the characterization of brain functions on the basis of finely resolved maps of the brain.

## 1 Introduction

A wide variety of gray matter regions and white matter connections of the brain interact in order to produce complex movements. Specifically, the motor system includes gray matter structures, such as the premotor and motor cortices, basal ganglia, the cerebellum, areas of the association cortex and portions of the thalamus, as well as white matter bundles, such as the corticospinal tract (traditionally considered the principal mediator of voluntary movements), the vestibulospinal, reticulospinal, rubrospinal and tectospinal tracts (Nolte, 2009).

A frequently used technique for defining the anatomical correlates of functions in stroke patients is voxel-based lesion-symptom mapping (VLSM) (Bates et al., 2003). This method indicates the univariate association of damaged voxels with a particular deficit. Previous studies showed that VLSM maps obtained, for instance, for speech fluency and language comprehension (Bates et al., 2003), as well as such derived for the orienting of attention (Toba et al., 2017; Verdon et al., 2010) were in agreement with findings from functional brain imaging. However, several studies have emphasized that VLSM is sensitive to the distribution of lesions within vascular territories and the frequency of impaired voxels (Mah et al., 2014; Arnoux et al., 2018), emphasizing the need to use newly developed lesion inference approaches, particularly anchored in multivariate inferences (Karnath and Smith, 2014; Khalilian et al., 2024; Nachev, 2015). Specifically, such techniques are capable of defining and calculating the interrelated contributions of network elements from a dataset of multiple perturbations (or lesions) (Keinan et al., 2004a). Multivariate machine learning approaches, such as classification by support vector machines (SVMs), can be used to map brain functions onto cerebral structures (Corbetta et al., 2015; Forkert et al., 2015; Smith et al., 2013; Zavaglia et al., 2015; Zhang et al., 2014). As a further alternative, the Multi-perturbation Shapley value Analysis (MSA) represents a lesion inference approach based on game theory, designed to calculate the contribution of the network elements (specifically, brain regions) and the interactions existing between them, based on a dataset of multiple lesions. Brain regions are considered as “players” in a game who interact to achieve a behavioral outcome. This approach was validated in ground truth simulations as a better-performing option for lesion inference than VLSM (Zavaglia et al., 2024) and has already been applied to lesion inference in studying brain functions (Zavaglia et al., 2015; Malherbe et al., 2021) as well as specifically in the context of attentional functions (Kaufman et al., 2009; Zavaglia and Hilgetag, 2016; Malherbe et al., 2018; Toba et al., 2017; Toba et al., 2020c).

At the behavioral level, a very widely used measure of the motor function in stroke patients is the National Institute of Health Stroke Scale (NIHSS) (Brott et al., 1989). This scale is used to generally characterize the clinical or functional status of stroke patients and, to this end, regroups items testing different functions, such as the level of consciousness, horizontal eye movements, visual field, facial palsy, motor arm, motor leg, limb ataxia, sensory, language, dysarthria, extinction and inattention. NIHSS provides an efficient measure with strong clinical validity to quantify stroke severity. However, the detailed assessment of each function included in the NIHSS requires investigations of different functional elements and this aim cannot be accomplished with a global rating score such as the NIHSS. Because its validity in assessing deficits with known anatomy (such as motor function) has been demonstrated, the NIHSS has previously been used in new methods examining clinico-anatomical correlations (e.g., Arnoux et al., 2018; Menezes et al., 2007; Zavaglia et al., 2015; Malherbe et al., 2021). It has been shown that in the analyses conducted on behavioral results obtained with traditional scales, such as the NIHSS and the modified Rankin scale, a better prediction of stroke severity could be obtained only when considering both the volume and the lesion location obtained on structural magnetic resonance imaging (MRI) data (Forkert et al., 2015; Menezes et al., 2007). By analysing the global NIHSS and lesions of 148 acute stroke patients with a multivariate approach and focusing solely on gray matter structures, Zavaglia et al. (2015) inferred various locations underlying functions tested by the NIHSS, such as the bilateral caudate, left insula and bilateral parietal and frontal lobes. Of note in these results was the presence of bilateral frontal regions (also comprising primary and supplementary motor brain areas) and basal ganglia involved in the motor system, likely linked to the fact that motor symptoms result in high score values of the NIHSS (specifically, they can explain up to 18 of 42 possible score points). However, in order to more specifically explore motor functions, an individual sub-score of the NIHSS that focuses on motor tasks should be considered. Moreover, data available in Zavaglia et al. (2015) allowed only the analysis of functional inferences of gray matter structures while white matter connections should also be considered in order to completely characterize causal functional inferences in a given system (see also Toba et al., 2020a; Toba et al., 2020b; Godefroy et al., 1998). Malherbe et al. (2021) considered a high resolution parcellation of the brain into 294 white matter and gray matter regions in a large population of 394 acute stroke patients. These authors reduced the number of regions to only those that significantly inferred some brain functions (specifically the “left motor function,” “the right motor function” and the “language and consciousness function”) issued from NIHSS factors previously published by Lyden et al. (2004). Specifically, the VLSM approach was used to first reduce the regions to one hemisphere. Then, the study used an iterative loop performing MSA and discarding the region with the smallest contribution to a function. As a result, Malherbe et al. (2021) inferred for each function a base set of causally contributing regions. Concretely, the dorsolateral putamen and the posterior limb of the left and right internal capsule were related to the motor functions right and left, respectively. Whereas the left motor function was also associated with the superior corona radiata and the paracentral lobe of the right hemisphere as well as the right caudal area of the cingulate gyrus, the right motor function was related to the prefrontal gyrus, the external capsule and the sagittal stratum fasciculi of the left hemisphere.

In the present paper, by taking advantage of the well-characterized anatomical and functional model of the motor system and an improved estimated MSA algorithm, we aimed to explore causal functional contributions of both, gray matter structures as well as white matter connections, without any preselection of a subset of regions. To this aim, we used the newly developed approach of iterative estimated MSA in a large sample of 272 patients with motor impairments assessed by the NIHSS motor sub-score.

## 2 Materials and methods

### 2.1 Participants

We analyzed clinical behavioral and MRI data of 272 patients (mean age 64.1 years, SD 11.3, 59.6% male) included in the GRECogVASC study (NCT01339195) assessing post-stroke cognitive and motor status and its determinants in French-speaking patients (Arnoux et al., 2018; Barbay et al., 2018; Godefroy et al., 2012). All patients gave their written informed consent to participation. The study was performed in accordance with institutional guidelines and was approved by the regional investigational review board (*Comité de Protection de Personnes Nord-Ouest II*, Amiens, France; reference: 2010/25). Table 1 reports the demographic and clinical characteristics of the study cohort. We included patients presenting unilateral and bilateral strokes due to both cerebral infarct and hemorrhage, because hemorrhage may extend outside the arterial territories, and consequently, the association of both stroke subtypes decreases multicollinearity (i.e., data correlations among neighboring voxels) (Kreisler et al., 2000). All patients were assessed at 6 months post-stroke, using MRI and the NIHSS to quantify symptom severity in stroke (Brott et al., 1989). NIHSS is composed of 11 items concerning specific functional abilities (see section “1 Introduction”). By using the NIHSS motor sub-scale, we assessed the presence of hemiparesis, a deficit frequently used in clinico-anatomical correlations studies because of its known anatomical basis centered on the motor system, including the corticospinal tract and the precentral cortex (Godefroy et al., 1998; Kassubek et al., 2005; Zhu et al., 2010). To this aim, we derived a global limb motor score able to quantify the motor deficit linked to dysfunction in the motor system (the facial paresis score was not included, as we focused on limb paresis). The score corresponded to 10 minus the sum of the upper and lower limb items in the NIHSS; a score of 10 corresponded to no impairment in the limbs motor system, whereas a score of two indicated major impairment. Left hemiparesis was defined by a left limb motor score < 10. We restricted our analyses to the left motor score, as the motor deficit was more frequent on the left side (*n* = 30) than on the right side (*n* = 24) (Table 1).

**TABLE 1:** Demographical and clinical characteristics of patients included in the study.

	Patients (*n* = 272)
Age (years, mean ± SD)	64.1 ± 11.3
Male (%)	59.6
Handedness (right/other) (%)	92.3/7.7
Stroke subtype (infarct/hemorrhage) (%)	90.1/9.9
Post-stroke delay (days) (mean ± SD)	174 ± 18
Lesion side (Left / Right / Bilateral) (%)	27.6/33.8/38.6
First / Recurrent stroke (%)	81.6/18.4
NIHSS 6 months (mean ± SD)	1.71 ± 2.8
Motor score: right/left (mean ± SD)	9.82 ± 0.66/9.64 ± 1.29
Rankin (0/1/2/3/4) (%)	19.1/24.6/20/27.9/8.4
Antidepressant treatment (%)	18.75

Expressed as percentage (%) or mean ± standard deviation (SD).

### 2.2 Neuroimage processing

**FIGURE 1:**
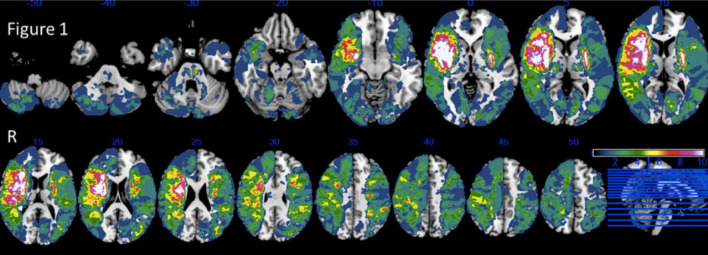
Lesion overlap of 272 patients. Reprinted from Neuropsychologia, 121/ 218, Arnoux, A., Toba, M.N., Daouk, J., Constans, J.-M., Puy, L., Diouf, M., Barbay, M., Godefroy, O., Is VLSM a valid tool for determining the functional anatomy of the brain? The need for an additional multivariate step, 69–78, Copyright (2025), with permission from Elsevier.

**FIGURE 2:**
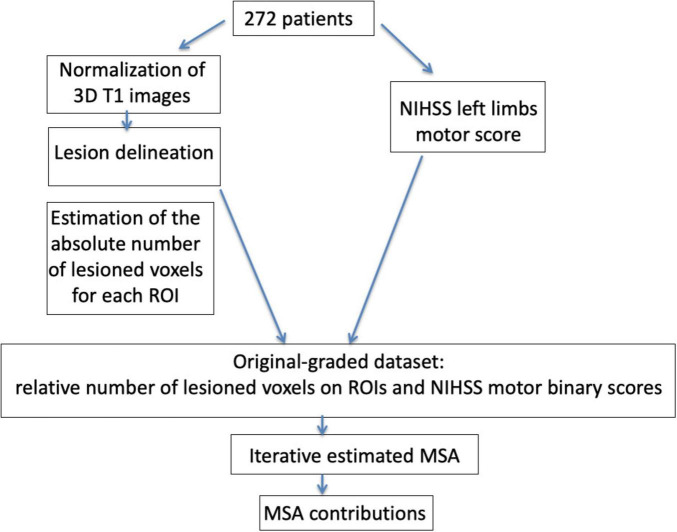
Schematic representation of the methodological approach of the study. Magnetic resonance imaging (MRI) images of 272 patients were normalized on a template adapted for older individuals (Rorden et al., 2012) and lesions were then delineated. In order to prepare the data for the application of the Multi-perturbation Shapley value Analysis (MSA), an estimation of the absolute and relative number of lesioned voxels was performed on the regions of interests (ROIs) used in the analyses. We generated the original-graded dataset [272 patients, 150 ROIs and one ROI designing the rest of the brain (RoB)] and then applied to random forest classifier in order to compute the performance scores [inverse binary motor National Institute of Health Stroke Scale (NIHSS) scores]. Finally, the iterative estimated MSA approach included the computation of ROIs contributions and was conducted until we found the smallest set of regions with a negligent contribution of the rest of the brain. ROI, region of interest; MSA, Multi-perturbation Shapley value analysis.

Magnetic resonance imaging scans included high-resolution T1-weighted images (inversion-recovery ultrafast gradient echo with magnetization preparation; fields of view (x, z): 205, 256; acquisition matrix (x, z): 256, 512; voxel size (x, y, z): 0.8 mm, 2 mm, 0.5 mm; sequence parameters: TR/TE: 13/4.5 ms; inversion time: 400 ms; flip angle: 15°; receiver bandwidth: 20.83 kHz; acquisition time: 3 mn 28 s), FLAIR, T2 and T2*-weighted sequences obtained at 6 months after stroke on a 3T machine (HDXt, General Electric Medical System) equipped with an eight-channel head coil. MRI datasets were registered into a template for older individuals (Rorden et al., 2012), using the pyramidal block-matching Diffeomorphic Demons algorithm (Garcia et al., 2010) implemented in the MedINRIA software package^1^ (Toussaint et al., 2007). Lesion delineation was performed manually (by trained neurologists: M.B., A.A., O.G.) on 3DT1 MRI datasets by applying the levelset algorithm in Medical Image Processing, Analysis and Visualization software (Covington et al., 2010). Lesions were defined as cavitations on T1 sequence. Normalized lesioned brain structures (further called regions of interest, ROIs) were determined using NiiStatV9 (Rorden et al., 2007) and Automatic Anatomical Labelling (AAL) (Tzourio-Mazoyer et al., 2002) and NatbrainLab (Catani and Thiebaut de Schotten, 2008) templates, representing both gray and white matter structures (see Figure 1) and put together in the AALCAT atlas. The entire methodological pipeline is summarized in Figure 2.

### 2.3 Multi-perturbation Shapley value Analysis (MSA)

#### 2.3.1 General MSA approach

As previously described (Kaufman et al., 2009; Keinan et al., 2004b; Zavaglia et al., 2015), the MSA approach assesses causal function localization from multiple perturbation data, based on coalitional game theory (Shapley, 1953). The system elements (here, the 150 areas from the AALCAT atlas, and a region representing the “rest of the brain,” RoB) can be seen as players in a coalition game. The RoB is computed in order to capture potential contributions of ROIs not included *a priori* in the analysis. For each configuration, the performance of the system is obtained. The aim of the analysis is to assign values, representing the ROIs’ contribution to, or importance for, overall (neural) function. The contribution value of a player, formalized as the Shapley value (Shapley, 1953), represents the difference between the worth of all coalitions that contain the element and the worth of all coalitions that do not contain it. For further information concerning the technical details and a more detailed description of the MSA see Keinan et al. (2004b).

#### 2.3.2 Data preparation for MSA - from original-graded dataset to complete-predicted dataset

For each patient of the dataset, the graded measure of relative lesion size of each ROI (that is, the percentage of lesioned voxels within each of the 151 ROIs, from zero to 100% of lesion) was associated with the binarized motor NIHSS score. Specifically, motor NIHSS scores equal to 10 were considered “normal” (1), whereas scores smaller than 10 were considered “pathological” (0). Since the binary scores represent the severity of neurologic *deficit*, while MSA requires a score representing behavioral *ability*, we used the inverse of each score as an indicator of functional performance (1 *– current score*).

We then computed *pairwise* Pearson correlation coefficients using MATLAB (Mathworks Inc., Natick, United States^2^) for the relative regional lesion patterns (i.e., correlations between all pairs of relative lesion sizes), across the 151 ROIs.

In order to characterize the contribution of each ROI for the motor NIHSS values, and to find the smallest set of ROIs with a negligent contribution of the RoB, we used the iterative estimated MSA approach. The dataset, composed of 272 graded lesion configurations (describing relative lesion size) for 150 AALCAT brain regions and the RoB as well as the corresponding performance scores did not represent the full set of possible combinations of binary states of the *N* = 151 ROIs, as is typical for opportunistic samples (*original-graded dataset*). In this study, a total of 2*^N^* of binary lesion configurations would have to be generated for an exhaustive analysis, which was not practically possible. Therefore, we used the approach of an estimated MSA analysis. Specifically, the MSA was run iteratively until the smallest set of ROIs contributions to motor deficits with a non-significant contribution of the RoB was found (Malherbe et al., 2021). The estimated Shapley value is calculated on a random sample of permutations (selection of a set of multi-perturbation configurations), whose performance should be measured. Here we chose 1,000 permutations for 151 regions. Since this is an unrealistic scenario for available data, a machine-learning-based approach, using random forest, was applied before MSA to estimate the set of clinical phenotypes according to the lesion pattern configurations (Malherbe et al., 2018; Malherbe et al., 2021; Zavaglia et al., 2015; Zavaglia et al., 2016). All analyses were performed using Python in-house scripts available here: https://github.com/ShreyDixit/MSA-App/. The analysis comprised three steps, as illustrated in the Figure 2:

(1) Optimization of the random forest parameters prior to MSA: From the graded matrix containing clinical and lesion data of all patients, random forest parameters were optimized. Random forest hyperparameter selection was based on the number of trees, the tree depth, the number of features, if we used bootstrap and minimum number of leaf and split. All possible combinations of these parameters were tuned for the datasets. Random forest parameters selection was based on F1-scores.
(2) Computation of an estimated MSA with a bootstrap procedure to ensure the robustness of the results: This step aimed to quantify the causal functional contributions of the ROIs for the motor score, by using an objective value characterizing the contributions of ROIs across all possible lesion configurations, the Shapley value (Shapley, 1953). We used the estimated MSA to derive the Shapley value (Keinan et al., 2006; Malherbe et al., 2021). The optimized random forest parameters from (1) were used in this step to define functional behavior related to a set of configurations needed in the estimated MSA procedure. To ensure the robustness of the obtained contributions and to define the standard error, we performed 1,000 samples of bootstrapping the estimated MSA approach with 1,000 permutations. Specifically, from the available database, we chose 1,000 random samples with replacements, with the size of the original dataset. We then performed the estimated MSA on each of these 1,000 new bootstrap samples (with the size of the original dataset). Finally, the functional contributions and standard error of each ROI were averaged across the 1,000 samples.
(3) Discarding of the ROIs with the smallest contribution to behavior and those with a negligible amount of contribution to behavior, and update of the RoB accordingly (by adding the discarded ROIs in the RoB).

Steps 1–3 were repeated until we found the smallest set of ROIs with a non-significant contribution of the RoB [refer to Malherbe et al. (2021) for more details]. This set of ROIs was considered to comprise brain regions causally contributing to motor deficits.

We assessed the classifier performance of the predictor by computing a *classification (prediction) accuracy* applying a “leave-one-out” cross-validation on the original-graded dataset for every test, using in turn each single case from the training data as the *validation data* and all the remaining cases as the *training data*. Specifically, classification accuracy was computed by counting the number of successful predictions (i.e., the number of times that the real binary score was predicted correctly) in the “leave-one-out” cross-validation. In this procedure, a value of 100% indicated that the random forest predicted correctly the scores for all the ∼272 clinical cases. The result of this process yielded a prediction classification accuracy of 95%. The accuracy level was substantially and significantly higher than the statistical chance level (80%). The statistical chance level was performed by using the true binary motor score that we shuffled across all patients to obtain the performance of a totally arbitrary classifier (by chance) on the data. As an additional measure of the performance of the binary prediction, we computed the *F1-score.* This estimate is computed as $F\,1-s\,c\,o\,r\,e=\frac{2^{*}\,P\,r\,e\,c\,i\,s\,i\,o\,n*R\,e\,c\,a\,l\,l}{P\,r\,e\,c\,i\,s\,i\,o\,n+R\,e\,c\,a\,l\,l}$, with $P\,r\,e\,c\,i\,s\,i\,o\,n=\frac{T\,P}{T\,P+F\,P}$ and $R\,e\,c\,a\,l\,l=\frac{T\,P}{T\,P+F\,N}$, with TP: true positive, FP: false positive, TN: true negative. A value of 1 indicates a perfect precision and recall in the prediction, whereas a value of 0 indicates that precision and recall have a value of 0. The F1-score for the inverse motor NIHSS was 0.88. We summarize the entire methodological pipeline in Figure 2.

## 3 Results

**FIGURE 3:**
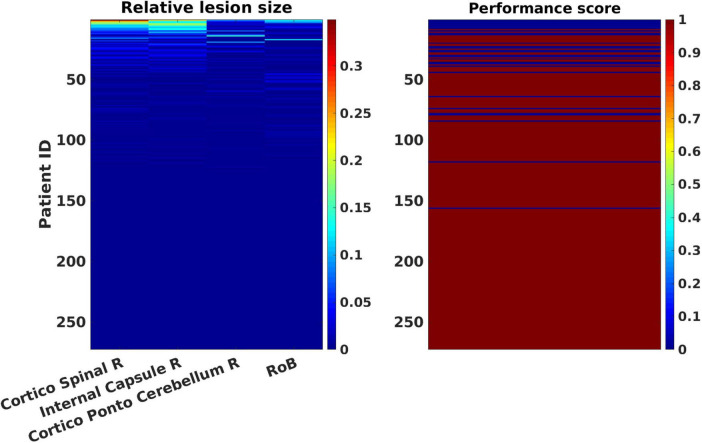
Patterns of relative lesion sizes of positive contributors and associated binary motor National Institute of Health Stroke Scale (NIHSS) values in the patients’ sample. The left panel represents the relative lesion size [in % of damaged voxels with respect to the total number of voxels for each regions of interest (ROI)] for the most lesioned ROIs, plus one additional region representing the “rest of the brain,” RoB in the 272 patients’ sample. Relative lesion patterns are associated with inverse binary motor NIHSS scores (right panel). Patient cases are shown sorted in descending order from largest to smallest lesion sizes. The color-coded scale displays the relative lesion size (from 0, in blue hues, to 100% of lesioned voxels in red hues). The binary motor inverse NIHSS values of the clinical tests are represented in blue (0: “pathological”) or dark red (1: “normal”) in the rightmost color-coded scale.

Figure 3 displays patterns of relative lesion sizes of positive contributors and associated binary motor NIHSS values for the 272 patients. Performance scores were associated with different sizes of lesion in the set of selected ROIs. Consequently, patients with large lesions as well as patients with small lesions in a given ROI could present pathological scores.

**FIGURE 4:**
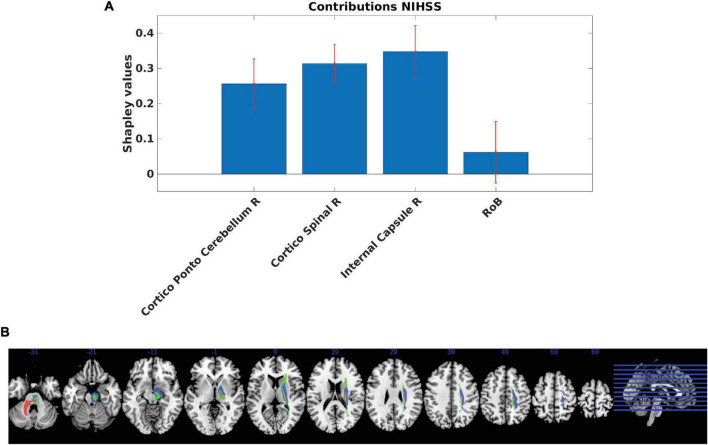
**(A)** Regional Multi-perturbation Shapley value Analysis (MSA) functional contributions to motor function. Smallest set of regions with a negligent contribution of the rest of the brain (RoB). Estimated MSA contribution values (+/− standard deviation, SD) provided by the iterative estimated MSA method computed using the original-graded dataset based on the random forest prediction of performance scores. The contributions and standard deviations were derived from the average of 1,000 random samples with replacement (bootstrap approach). Positive values indicate positive contributions (hence injury of the respective regions leads to decreased performance). Most of the contributions were statistically significant (except for the RoB). **(B)** Anatomical representation of regional MSA contributions to motor function. MSA contributions represented in brain regions defined by the AALCAT atlas. Red color represents the Cortico-Ponto-Cerebellum Right Tract, blue color represents the Cortico-Spinal Tract Right and the green color represents the Internal Capsule Right. Results are illustrated on a brain template in MNI standard space oriented in neurological convention (right hemisphere on the right side). MNI coordinates of each section (z-axis) are shown.

Our analyses showed that the internal capsule, the corticospinal tract and the cortico-ponto-cerebellum bundles in the right hemisphere made the strongest positive contributions (Figure 4). The MSA contribution values were significantly different from 0, except for the RoB. In line with the employed methodology, the RoB did not present a significant contribution. Positive contributions indicate that a region or a set of regions supports the performance in a given clinical test. Thus, if these regions were injured, performance would decrease. By contrast, a negative contribution indicates that a region hinders performance and implies that damage of the region may actually improve clinical performance scores in brain-damaged patients. However, one should note that the lesion of negative contributors might improve a given function only in patients with specific lesional patterns, without being generalizable to all brain-damaged patients. Supplementary Figures 1, 2 present the same analyses performed by leaving out the patients with bilateral and recurrent strokes.

**FIGURE 5:**
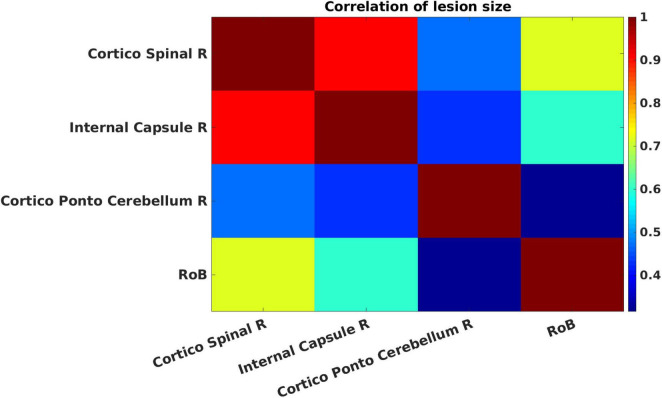
Cross-correlations of lesion patterns across the positive contributors for the 272 patients. The strength of the Pearson correlation of pairs of regions of interest (ROIs) is color-coded from low (blue) to high (red). All correlations are statistically significant (*p* < 0.05).

Figure 5 presents cross-correlations of lesion patterns across the positive contributors for the 272 patients. All correlations were statistically significant (*p* < 0.05). These results allowed us to assess the covariance of lesion patterns across ROIs, which could be caused by their dependence on a common source of blood supply (i.e., co-localization within the same vascular territory). The results obtained for regions that have largely independent lesion patterns (i.e., showing correlation between lesion sizes being lower than 0.5), are important for the interpretation of genuine functional overlap indicated by redundant functional interactions. A strong correlation was observed between the cortico-spinal tract and the internal capsule. Weaker correlations were observed between the cortico-spinal tract and the cortico-ponto-cerebellum bundle, and between the internal capsule and the cortico-ponto-cerebellum bundle.

## 4 Discussion

The aim of the present study was to use a multivariate (estimated MSA) approach in order to quantify functional contributions of both gray matter regions and white matter connections in motor function. To address this issue, we analyzed a sizable cohort of 272 stroke patients and evaluated their motor NIHSS values in conjunction with the brain lesion patterns. Our results showed that MSA was able to quantify different motor contributors, particularly emphasizing the roles of the corticospinal tract, the internal capsule and the cortico-ponto-cerebellum bundle as positive contributors. No negative contributions were highlighted. As expected, the RoB contribution was not significant.

### 4.1 MSA results

The present study reflects the iterative estimated MSA, a novel MSA-based approach to characterize contributions of both gray matter structures and white matter connections simultaneously in a given function. A similar method has been used by Malherbe et al. (2021) in combination with VLSM, which allowed to pre-select a subset of regions in order to reduce the number of regions for the subsequent MSA. Due to methodological advances in computing the MSA, in the present study no preselection of ROIs needed to be performed. This advance allowed us to test directly the ability of the iterative estimated MSA approach for making causal functional inferences in motor function based on brain lesion deficits.

The MSA quantified relative contributions of both gray matter structures and white matter connections. Positive contributions obtained with MSA emphasized the roles of the internal capsule, corticospinal tract and cortico-ponto-cerebellum as principal contributors in motor function. First, the key roles of the corticospinal tract and internal capsule confirm previous findings reported in the motor function analysis by using alternative methods (Arnoux et al., 2018; Godefroy et al., 1998; Kassubek et al., 2005; Malherbe et al., 2021; Zhu et al., 2010) and emphasize the importance of white matter connections. These results suggest that the iterative MSA was able to rank positive contributors when jointly analysing gray matter structures and white matter connections as previously described (Kaufman et al., 2009; Malherbe et al., 2021; Ofir-Geva et al., 2023). Second, we inferred contributions of structures that had not been evident as playing a key role in motor deficit (Corbetta et al., 2015; Karnath and Rennig, 2017; Rorden et al., 2009). These structures involved the cortico-ponto-cerebellum bundle as a positive contributor. This pathway originates in the motor cortex, the prefrontal and temporal cortices (Salmi et al., 2010). The characterization of the cortico-ponto-cerebellum white matter network functional role in human is still challenging, but current findings suggest an involvement in both motor and cognitive functions (Palesi et al., 2017), although we cannot exclude that multicollinearity might also account for the present association.

We should note that the contribution of the RoB was non-significant in our MSA analyses. Specifically, the contribution of the RoB is a strong indicator of the absence of other potential structures involved in motor function that would have not been considered as regions of interest in the analyses. Of note, an important RoB contribution would indicate that the analyzed function is dependent on brain regions potentially missing from the analysis. However, we should note that the RoB reflects the different lesion patterns of many distinct regions. Small but critical regions may become “diluted,” preventing the RoB from reaching a significant level (Ofir-Geva et al., 2023). Alternatively, it can be hypothesized that the lack of a RoB contribution could be related to the inconsistency of lesion locations and to the weak signal of lesion load in regions that are functionally related to the deficit. As a result, this signal cannot be of much utility to the model. While this explanation is also possible, one should consider the substantial size of 272 patients’ sample in the present study, limiting such alternative explanations. Furthermore, the F1-score (considering recall and precision in the analyses) was very high (0.88).

It could be argued that regions such as the precentral gyrus (primary motor cortex) should also be involved in the motor function. Given that the present study has been conducted in chronic patients, it could be that the presence of lesions in these regions in the acute stage is potentially compensated by an intact corticospinal tract or internal capsule. Thus, lesions inducing temporary motor impairment resolve after the acute stage (Karnath and Rennig, 2017) and this may account for the absence of an independent effect of precentral lesions (el Quessar et al., 1999; Godefroy et al., 1998). Other possible explanations include the fact that isolated lesions in the primary motor cortex often recover in the chronic phase, leading to impaired dexterity that the NIHSS does not assess. Additionally, the combined NIHSS sub-scores for upper and lower limbs may dilute the signal, as the primary motor cortex may play a lesser role in lower limb function. Finally, this outcome might be due to an insufficient representation of primary motor cortex lesions in the cohort (10 out of 272 patients presented lesions of the superior corona radiata, including premotor and primary motor cortices lesions), which can occur even in large study populations.

### 4.2 MSA and brain-behavior relationships

Multi-perturbation Shapley Analysis was previously used in order to define brain-behavior relationships. Specifically, MSA was initially used in datasets from cooling deactivation experiments and permanent lesion experiments (e.g., Keinan et al., 2004b; Zavaglia et al., 2016) and results proved to be robust in ground truth simulations (Zavaglia et al., 2024). The plausibility of applying MSA on lesion data was also demonstrated in studies conducted in cohorts of stroke patients with visuospatial neglect (Malherbe et al., 2018; Toba et al., 2017; Toba et al., 2020c) that confirmed and specified contributions of fronto-parietal and occipital systems to visuospatial attentional behavior (Corbetta et al., 2008; He et al., 2007; Hilgetag et al., 2001; Kincade et al., 2005; Thiebaut de Schotten et al., 2005; Toba et al., 2018; Verdon et al., 2010; Toba et al., 2022; Toba et al., 2024; Kaufmann et al., 2025). However, caution is needed when interpreting MSA results. While MSA was proposed to address lesion-dependencies, this assumption holds mainly in theoretical scenarios where complete empirical data is available. In practice, MSA heavily depends on predictors and classifiers which can be biased by interdependencies within the data.

Generally, MSA results have been interpreted in the framework of inter-hemispheric rivalry theories (Keinan et al., 2004a; Keinan et al., 2004b) and find applications in studies conducted with non-invasive methods able to stimulate or disrupt different brain structures (see for details Valero-Cabre et al., 2019). Specifically, positive contributors (such as the corticospinal tract, the internal capsule, the cortico-ponto-cerebellum tract) represent structures that facilitate the performance and thus a clinical goal may be to enhance them. However, interpretations are more complex, because brain structures have intrinsic dynamics that allow them to compete for processing resources in order to accomplish brain functions. As a result, they may be at the same time positive and negative contributors in related functions.

### 4.3 Limitations

Several limitations of the present study should be discussed. First, our analyses purposely concerned a well-known functional system (the motor system). While this choice allows to discuss the efficacy of the iterative estimated MSA in identifying important motor regions, it also limits the novelty of our results. Moreover, the use of a relatively coarse measure for motor function (i.e., NIHSS upper and lower limb scores), even in a large cohort, is not ideal for contributing novel biological insights. More precise measures should provide a finer-grained understanding of the motor system’s condition.

Second, the lesion-based approach used in this study is not the definitive gold standard for computing lesions-symptom mapping analyses. However, motor function is among the best-understood functions in terms of functional anatomy. For this reason, we chose to use it as a model to evaluate our methodological approach.

Third, the train-set to predict the behavior was performed with a graded database, whereas the test-set was binarized. This choice respects the need to use different train and test set of data but also involves that the model performance should be treated with caution.

Another limitation of this study concerns the choice of ROIs in MSA. ROIs are defined anatomically but not functionally. As a result, chosen ROIs can combine different functional units and thus the computation of a lesion load may not be entirely informative on the functional role of the region.

Last, another limitation of this study is represented by the inclusion of patients with a history of prior strokes and bilateral strokes, potentially confounding the results. However, additional analyses emphasized only minor results changes when these patients were left out.

## 5 Conclusion

In summary, in the present study we successfully assessed the possibility of using the iterative estimated MSA in order to specify the contribution of gray matter structures and white matter connections in a well-known functional system. Based on these results, we argue that lesion inference approaches based on MSA are suited to contribute further to defining the functional neuroanatomy of the human brain.

## Data Availability

The raw data supporting the conclusions of this article will be made available by the authors, without undue reservation. The conditions of our ethics approval do not permit public archiving of full data. Readers seeking access to full data should contact the author OG at the Department of Neurology, University of Picardy. Access will be granted to named individuals in accordance with ethical procedures governing the reuse of sensitive data. Specifically, requestors must meet the following conditions to obtain the data (completion of a formal data sharing agreement).

## Footnotes

1
http://med.inria.fr/

2
www.mathworks.com/matlabcentral
