# Supplementary material for: Analysis of clinical anatomical correlates of motor deficits in stroke by multivariate lesion inference based on game theory
Source: Front Neurosci. 2025 Apr 17;19:1409107. doi: 10.3389/fnins.2025.1409107 (PMC12043593; doi:10.3389/fnins.2025.1409107)

Supplementary material

Fig. S1. Regional MSA functional contributions to motor function in a sample of 167 patients with unilateral lesions (extracted from the main sample of the study, n = 272). Smallest set of regions with a negligent contribution of the RoB. Estimated MSA contribution values (+/- standard deviation, SD) provided by the iterative estimated MSA method computed using the original-graded dataset based on the random forest prediction of performance scores. The contributions and standard deviations were derived from the average of 1000 random samples with replacement (bootstrap approach). Positive values indicate positive contributions (hence injury of the respective regions leads to decreased performance). Most of the contributions were statistically significant (except for the RoB). Given the substantial size of the removed sample, results changed accordingly and emphasize now also some other regions, presumably highlighting false positives errors (such as for the inferior fronto-occipital fasciculus) linked to a small sample size.

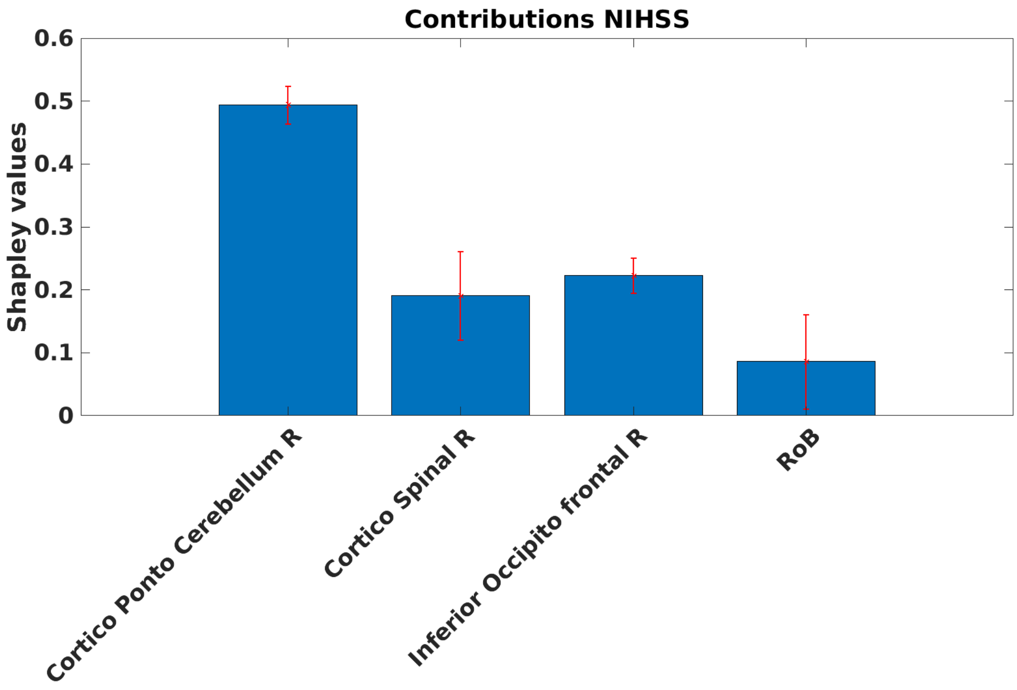

Fig. S2. Regional MSA functional contributions to motor function in a sample of 222 patients with a single stroke (extracted from the main sample of the study, n = 272). Smallest set of regions with a negligent contribution of the RoB. Estimated MSA contribution values (+/- standard deviation, SD) provided by the iterative estimated MSA method computed using the original-graded dataset based on the random forest prediction of performance scores. The contributions and standard deviations were derived from the average of 1000 random samples with replacement (bootstrap approach). Positive values indicate positive contributions (hence injury of the respective regions leads to decreased performance). All the contributions were statistically significant. Given the size of the removed sample, results changed slightly but the main positive contributions are similar to those obtained in the analyses of the whole sample of n = 272.

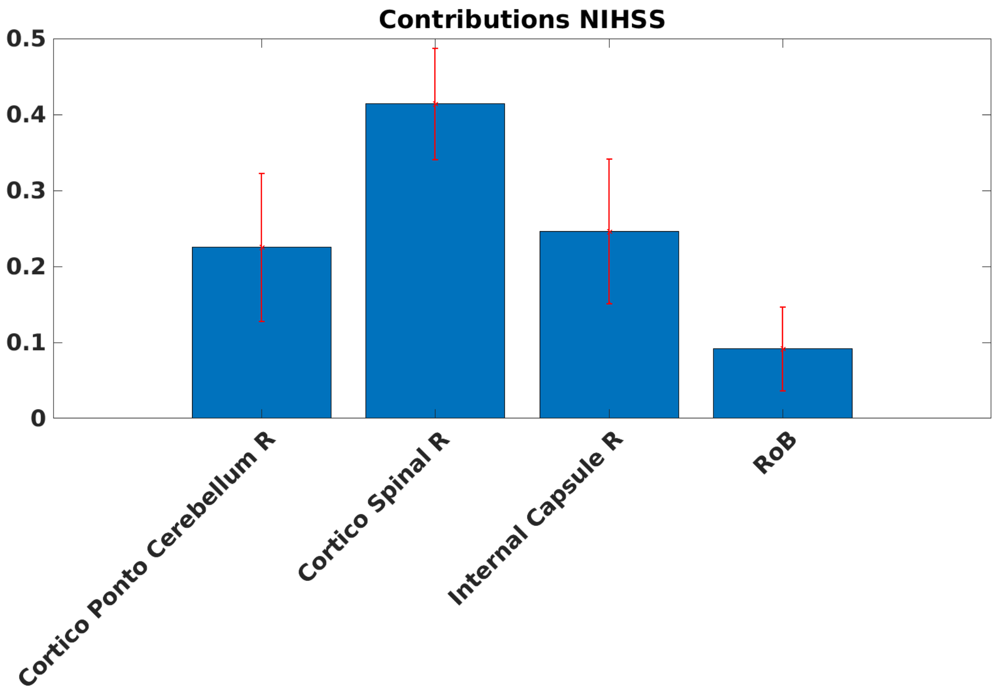

Supplement: Supplementary file 1 [file Data_Sheet_1.pdf]
